# Dasatinib demonstrates efficacy in organoid derived paclitaxel-resistant *Trp53/Cdh1-*deficient mouse gastric adenocarcinoma with peritoneal metastasis

**DOI:** 10.1186/s13619-025-00232-2

**Published:** 2025-04-29

**Authors:** Wenshuai Liu, Lingmeng Li, Leilei Guo, Haojie Li, Zhaoqing Tang, Xuefei Wang, Liyu Huang, Yihong Sun

**Affiliations:** 1 Department of General Surgery, Zhongshan Hospital, Fudan University, Shanghai, 200032 China; 2 Gastric Cancer Center, Zhongshan Hospital, Fudan University, Shanghai, 200032 China; 3 Retroperitoneal Sarcoma Center, Zhongshan Hospital, Fudan University, Shanghai, 200032 China; 4 Key Laboratory of Systems Biomedicine (Ministry of Education) and Collaborative Innovation Center of Systems Biomedicine, Shanghai Center for Systems Biomedicine, Shanghai Jiao Tong University, Shanghai, 200240 China

**Keywords:** Mouse model, Peritoneal metastasis, Diffused gastric cancer, ECM‒receptor interaction, Paclitaxel resistance

## Abstract

Gastric cancer peritoneal metastasis (GCPM) typically indicates a poor clinical prognosis and is frequently observed in diffuse gastric cancer (GC) patients with *CDH1* loss of function. GCPM characterized for its aggressiveness and resistance to chemotherapy, most notably paclitaxel (PTX), poses significant treatment challenges. Previously, no mouse gastric adenocarcinoma (MGA) cell lines with *Trp53* (encoding mouse p53) and *Cdh1* (encoding mouse E-cadherin) mutations and a high potential for peritoneal metastasis in mice have been established. Here, we derived a mouse GC cell line, called MTC, from subcutaneously transplanted mouse *Trp53*^*−/−*^*Cdh1*^*−/−*^ GC organoids. Through matching the short tandem repeat profile of MTC with those in current cell banks, we verified the uniqueness of MTC. Furtherly, we confirmed the features of MTC by detecting the expression of p53, E-cadherin, and pan-CK. After long-term exposure of the original MTC line to PTX, we developed a more aggressive, PTX-resistant cell line, termed MTC-R. Compared with MTC, MTC-R demonstrated enhanced tumorigenicity and high potential for peritoneal metastasis in subcutaneous and intraperitoneal tumour models both in BALB/c nude mice and C57BL/6 J mice. Transcriptome analysis revealed the ECM‒receptor interaction pathway activation during the development of PTX resistance, and dasatinib (DASA) was identified as a potential drug targeting this pathway. DASA showed promise in ameliorating disease progression and improving overall survival in MTC-R GCPM model in C57BL/6 J mice. Overall, we established a novel MGA cell line with *Trp53* and *Cdh1* mutations and its PTX-resistant variant and demonstrated the efficacy of DASA in treating PTX-resistant GCPM.

**Supplementary Information:**

The online version contains supplementary material available at 10.1186/s13619-025-00232-2.

## Background

Gastric cancer (GC) is defined as a primary malignant epithelial tumour originating from the stomach and is characterized by complexity and heterogeneity. Despite declining trends in incidence and mortality in some countries over recent decades, GC remains the third most common cancer in terms of both incidence and mortality in China. Globally, it accounts for nearly 44.0% of new GC cases and is responsible for 48.6% of GC-related deaths (Sung et al. 2021). In China, a significant proportion of new GC cases are diagnosed at advanced stage. Approximately 15% of newly diagnosed GC patients exhibit peritoneal metastasis (PM), and among those who undergo curative surgery, 50% experience recurrence with peritoneal spread (Allemani et al. 2018; Yonemura et al. 2006). Gastric cancer peritoneal metastasis (GCPM) is associated with a poor prognosis, often leading to death within 6 months of diagnosis (Mizrak Kaya et al. 2018). GC patients diagnosed with PM during the perioperative stage derive limited benefits from current antitumour therapy (Newhook et al. 2019; Saito et al. 2015; White et al. 2020). Thus, there is an urgent need to elucidate the molecular mechanisms underlying GCPM and explore new therapeutic strategies for these patients.

Many newly developed drug candidates have shown effectiveness in preclinical tests in immunodeficient mouse models, but fewer than 10% have been successfully applied in the clinic. In contrast to conventional chemotherapy, immunotherapy-based regimens usually lack a suitable preclinical mouse model for efficacy research. Spontaneous GC mouse models established via methods such as *N*-methyl-*N*-nitrosourea (MNU) drinking, *Helicobacter pylori* infection, or genetic engineering technology also have limitations, including time consumption (typically over 8 months) and the absence of PM (Han et al. 2002; Li et al. 2023). Organoids, which mimic parent tumour, and immune cell coculture systems represent a promising platform that is widely used for drug screening in immunotherapy. However, these coculture systems lack a complex tumour microenvironment and cannot be used to comprehensively evaluate drug efficacy in vivo (Gronholm et al. 2021; Magre et al. 2023). Therefore, establishing a transplanted GC tumour mouse model, especially a PM model in immunocompetent mice, would greatly facilitate the screening of targeted drugs and immunotherapeutic regimens for GC.

Yamamoto has developed stomach adenocarcinoma cell lines from C57BL/6 J mice induced by MNU in the drinking water (Yamamoto et al. 2018). These mouse GC cell lines have distinct tumorigenicity in terms of lung metastasis and intra-abdominal metastasis, characterized by a *Trp53* (encoding mouse p53) loss-of-heterozygosity background. According to the molecular classification of GC, subtypes with *CDH1* (encoding human E-cadherin) mutations are commonly associated with a poor prognosis (Cancer Genome Atlas Research, 2014; Cristescu et al. 2015). *CDH1* mutations uniquely occur in sporadic diffuse-type gastric cancer (DGC), which is particularly prone to PM and is associated with a poor prognosis.(Totoki et al. 2023) Recent advances in chemotherapy and chemoimmunotherapy in GCPM revealed that nonresponder patients presented increased frequencies of *TP53* (encoding human p53) and *CDH1* variations (Wang et al. 2020; Yu et al. 2024). Paclitaxel (PTX) is widely used in GCPM, yet screening drugs after PTX resistance occurs poses significant challenges. Hence, there is an urgent need to establish a mouse GC cell line with *Trp53* and *Cdh1* mutations and resistance to PTX.

Organoids, which represent an intermediate state between tissues and individual cells, have proven valuable for this purpose. Li et al. (2020) successfully established a colon signet ring cancer cell line derived from organoids, which inspired us. In our study, normal wild-type epithelial gastric organoids were previously isolated from *Trp53*^*flox/flox*^*;Cdh1*^*flox/flox*^*;Mist1*-CreER mice and induced to establish mouse GC organoids in vitro. We successfully isolated an expandable mouse gastric adenocarcinoma (MGA) cell line with loss of expression or function of *Trp53* and *Cdh1* from mouse GC organoid subcutaneous transplanted tumours. Furthermore, we enhanced the invasiveness of tumour cells by inducing resistance to PTX. We also assessed the tumorigenicity of the cell lines via a subcutaneous (s.c.) tumour model and an intraperitoneal (i.p.) model in BALB/c nude mice and C57BL/6 J mice. Finally, we investigated the mechanism underlying the discrepancy in PM potential between cells and revealed that dasatinib (DASA) tends to alleviate GCPM disease progression in a preclinical model.

## Results

### Isolation of cells derived from *Trp53/Cdh1*-deficient organoid-derived subcutaneous tumour

**Fig. 1:**
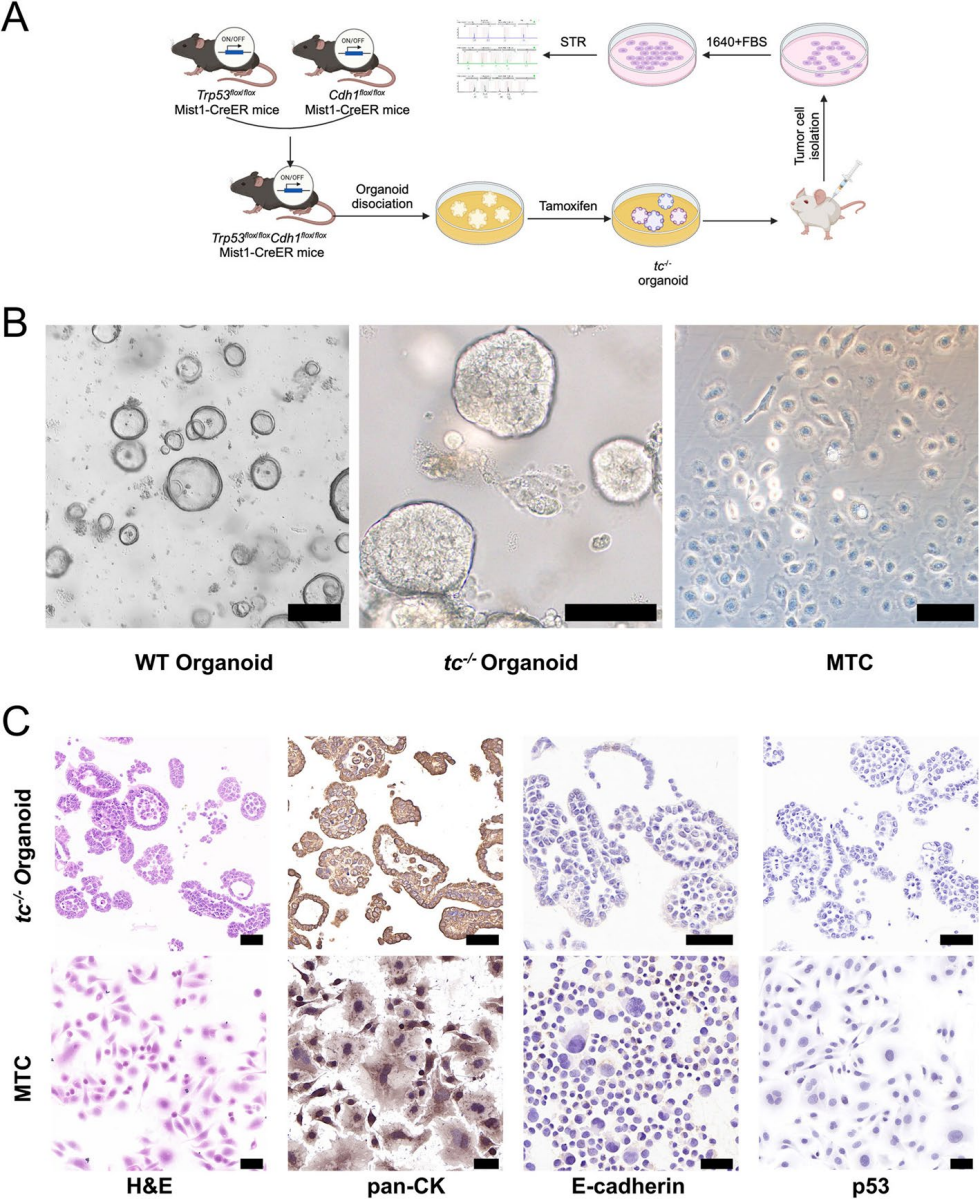
Generation and characterization of mouse gastric adenocarcinoma cells derived from organoids. **A** Schematic diagram of the method used to establish the mouse gastric adenocarcinoma cell line derived from *tc*^*−/−*^ organoids. **B** Microscopic morphology of WT organoids (scale bar, 400 μm), *tc*^*−/−*^ organoids (scale bar, 400 μm), and MTC cells (scale bar, 20 μm). **C** Representative H&E and IHC staining for pan-CK, E-cadherin, and p53 in the *tc*^*−/−*^ organoid and MTC cell lines. Scale bar, 50 μm. WT: Wild-type gastric epithelial organoid; *tc*^*−/−*^: mouse gastric cancer *Trp53*^*−/−*^*Cdh1*^*−/−*^ organoid

The process of establishing an organoid-transplanted s.c. tumour-derived MGA cell line is shown in Fig. 1A. Normal wild-type (WT) gastric epithelial organoids were isolated from *Trp53*^*flox/flox*^*;Cdh1*^*flox/flox*^*;Mist1*-CreER C57BL/6 J mouse and induced into *Trp53*^*−/−*^*Cdh1*^*−/−*^ (*tc*^*−/−*^) mouse GC organoids ex vivo. During this process, the WT organoids lost their large cystic glandular structures and tended to grow in solid cell clusters in *tc*^*−/−*^ organoids (Fig. 1B). Organoids were s.c. inoculated in the right flank of BALB/c nude mice. When the tumour volume reached 200 mm^3^, the s.c. tumours were harvested and digested into single cells. Through stepwise culture, the tumour cells got rid of the dependence on growth factors and the organoid niche. The tumour cells demonstrated as “fried egg” morphology and proliferated as individual cells resembling cell lines in 2D culture. Finally, the MGA cell line with *Trp53* and *Cdh1* mutations, namely MTC, was established through monoclonal selection (Fig. 1B).

Concurrently, similarity between MTC cells and *tc*^*−/−*^ organoids was also verified. The *tc*^*−/−*^ organoids exhibited an incomplete glandular structure, with cells showing multiple layers and atypia. H&E staining revealed that the MTC cells presented large nucleoli, intense staining, abnormal mitosis, and cell atypia (Fig. 1C). IHC assays revealed that both *tc*^*−/−*^ organoids and MTC cells were strongly positive for pan-CK, an epithelial-derived biomarker, indicating that their glandular epithelium originated. The expression of both E-cadherin and p53 was obviously detected in WT organoids (Fig. S1A), while absent in *tc*^*−/−*^ organoids and MTC cells (Fig. 1C). The WB and Sanger sequencing results verified the loss of *Trp53* or *Cdh1* in MTC cells and *tc*^*−/−*^ organoids (Fig. S1B, C). We further demonstrated the biological function of p53 protein in MTC cells. Nutlin-3, an inhibitor of p53 degradation, effectively suppresses p53 wild-type cancer cells by interfering with the degradation of p53.(Yan et al. 2018) Cell viability assay revealed that Nutlin-3 had no effect on MTC cells (Fig. S1D), which demonstrated that p53 loss of function occurred in MTC. The uniqueness of the MTC cells was also confirmed through STR profiling (detailed information is provided in Table S3). The STR profiles of MTC cells were compared against STR data from the ATCC, DSMZ, JCRB, and RIKEN databases, and there were no matches with the cell lines listed in these cell banks, confirming the uniqueness of the MTC cells. These results revealed that we established a novel MGA cell line, MTC, which originated from *tc*^*−/−*^ organoids and harbours both *Trp53* and *Cdh1* mutations.

### Establishment of paclitaxel-resistant MTC cells

**Fig. 2:**
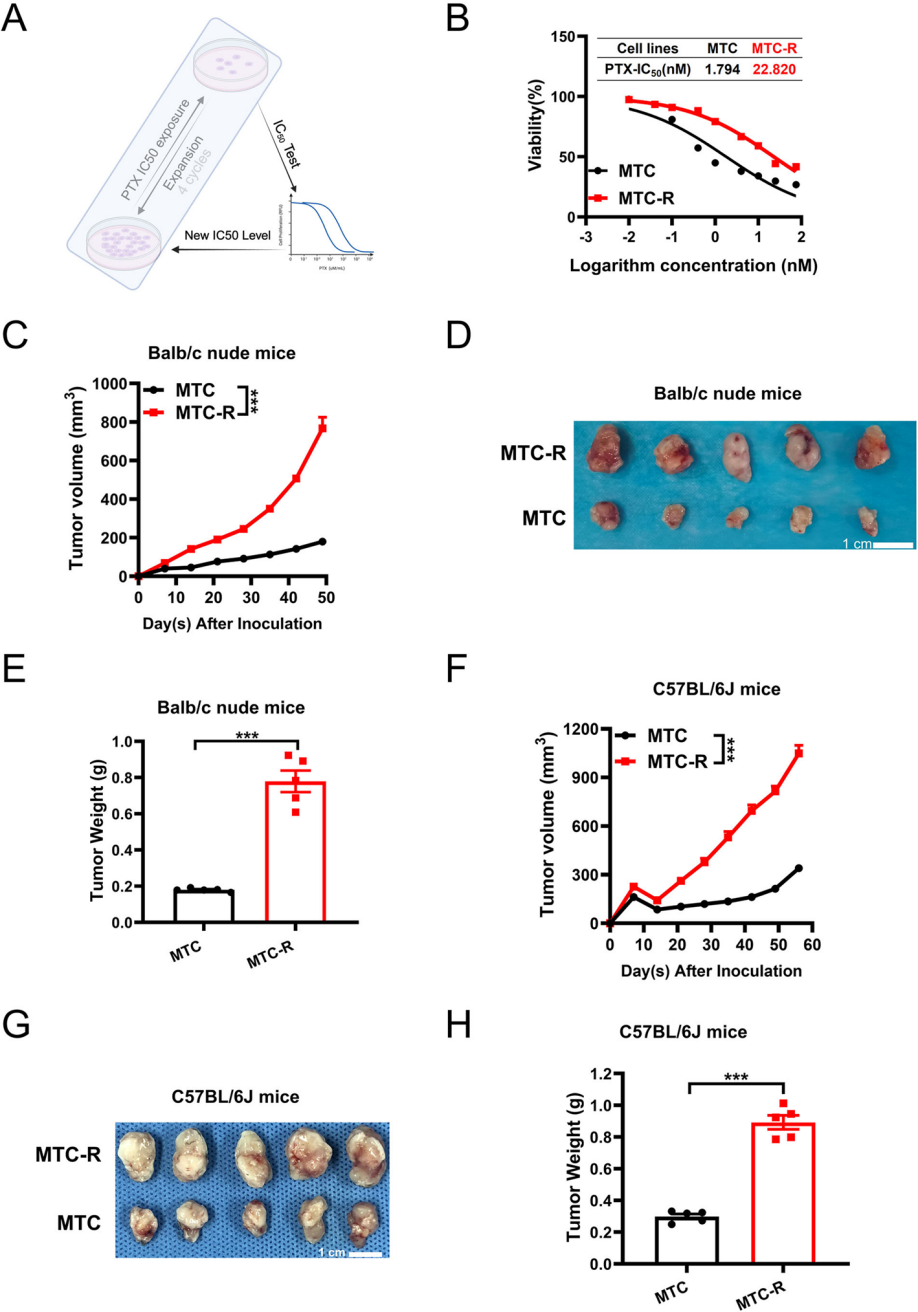
Generation of paclitaxel-resistant MTC (MTC-R) cells and their oncogenic features in a subcutaneous tumour model. **A** Schematic diagram of the establishment of a PTX-resistant MTC cell line named MTC-R. **B** IC_50_ values of MTC and MTC-R cells. **C** Curves of tumour volume in s.c. BALB/c nude mice. **D** Gross images of tumours after tumour resection at the endpoint of the experiment in the s.c. BALB/c nude mouse model. **E** Tumour weight in s.c. BALB/c nude mice at the endpoint of the experiment. **F** Curves of tumour volume in s.c. C57BL/6 J mice. **G** Gross images of tumours after tumour resection at the endpoint of the experiment in the s.c. C57BL/6 J mouse model. **H** Tumour weights of s.c. C57BL/6 J mice at the endpoint of the experiment. The data are shown as the mean ± SEM. ***: *P* < *0.001*; ns: not significant

PTX is widely used in various systemic or intraabdominal perfusion chemotherapies for GC, especially in the context of GCPM. However, selecting alternative drugs after PTX resistance represents a significant challenge in GCPM treatment. To investigate the mechanism and drug selection following PTX resistance, we generated a PTX-resistant MTC cell line, called MTC-R, by exposing the cells to PTX for more than 30 cycles (Fig. 2A), resulting in increased IC_50_ values ranging from 1.794 nM to 22.820 nM (Fig. 2B). The IC_50_ value of MTC-R was 12.720 times greater than that of MTC. Along with the occurrence of PTX resistance, MTC-R exhibited a spindle cell structure morphology (Fig. S1E). Consistently, compared with MTC cells, MTC-R cells also had significantly greater potential for proliferation (Fig. S1F). In vivo experiments revealed that the tumour volume (*P* < *0.001*) (Fig. 2C, D) and tumour weight (*P* < *0.001*) (Fig. 2E) in the MTC-R group were significantly higher than those in the MTC group in the s.c. BALB/c nude mouse model. The tumorigenicity of MTC and MTC-R cells in immune-competent mice, wild-type C57BL/6 J mice, was also assessed. Similarly, the MTC-R group presented a larger tumour size (*P* < *0.001*) (Fig. 2F, G) and greater tumour weight (*P* < *0.001*) (Fig. 2H) than those in the MTC group, demonstrating comparable robust malignancy potential in MTC-R. The mice bodyweight showed steady increased tandency, exhibited no significant difference between MTC and MTC-R group both in BALB/c nude and C57BL/6 J mice (Fig. S2A, B). Here, we established a PTX-resistant MGA cell line, MTC-R, derived from MTC. Both MTC and MTC-R showed differential potential for oncogenicity in s.c. immunodeficient and immune-competent mouse models. Moreover, we found that MTC-R exhibited strong malignant potential.

### MTC-R cell exhibited enhanced tumorigenicity in intraperitoneal metastasis tumour mouse model

**Fig. 3:**
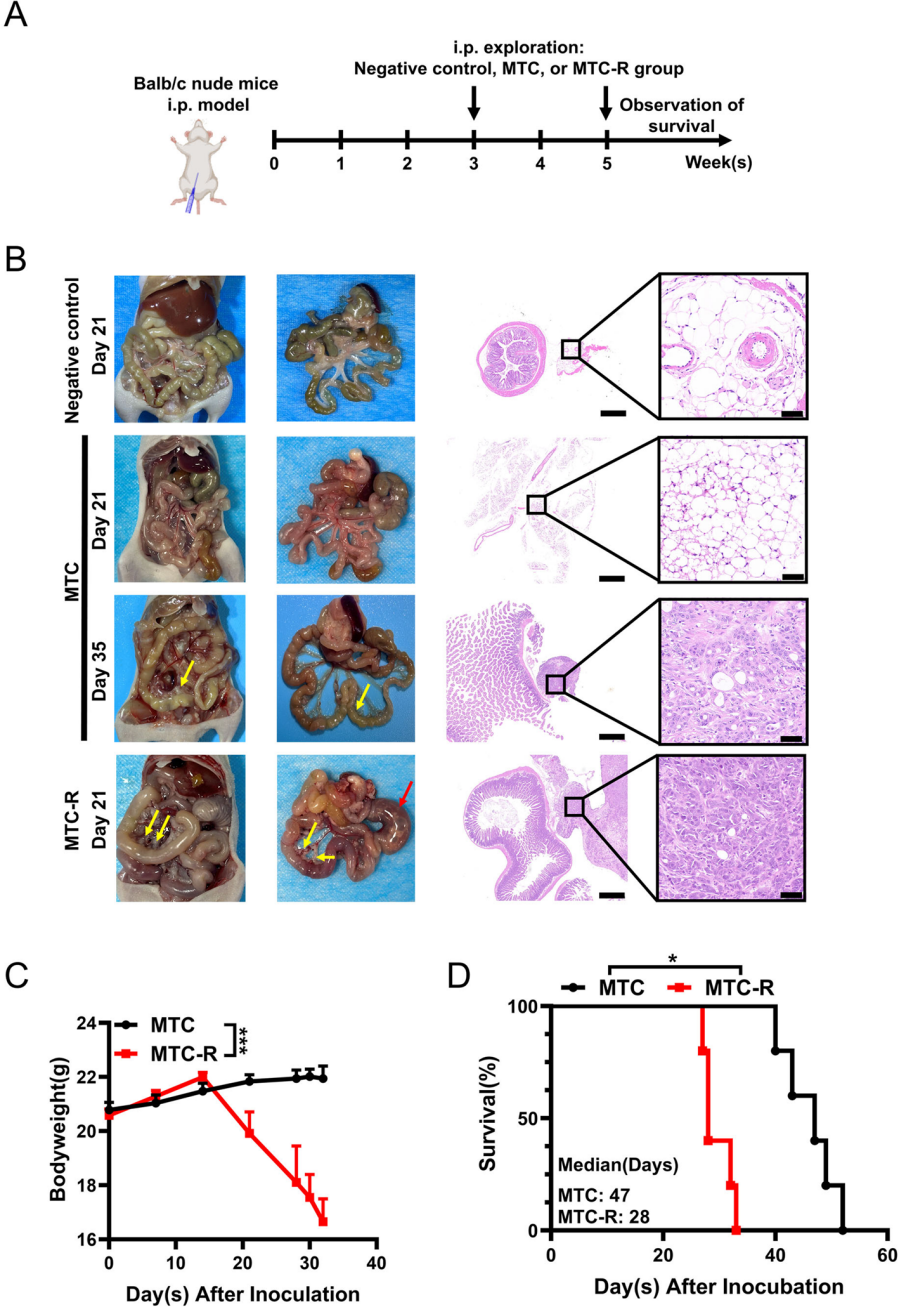
MTC-R cells exhibited enhanced tumorigenicity and aggressiveness in peritoneal metastasis tumour models in BALB/c nude mice. **A** Experimental scheme of the MTC and MTC-R i.p. model in BALB/c nude mice. **B** In the MTC group, obvious intra-abdominal metastasis was observed on day 21, whereas numerous peritoneal metastases (yellow arrow) were detected on day 35 after intraperitoneal inoculation in the MTC group. Mesenteric metastasis (yellow arrow) and intestinal obstruction (red arrow) were observed on day 21 after intraperitoneal inoculation in the MTC-R group. The scale bar of H&E staining was 500 μm (left) and 50 μm (right). **C** The body weights of the MTC and MTC-R i.p. model mice. **D** Overall survival significantly differed between the MTC and MTC-R i.p. models. The data are shown as the mean ± SEM. ***: *P* < *0.001*; *: *P* < *0.05*

**Fig. 4:**
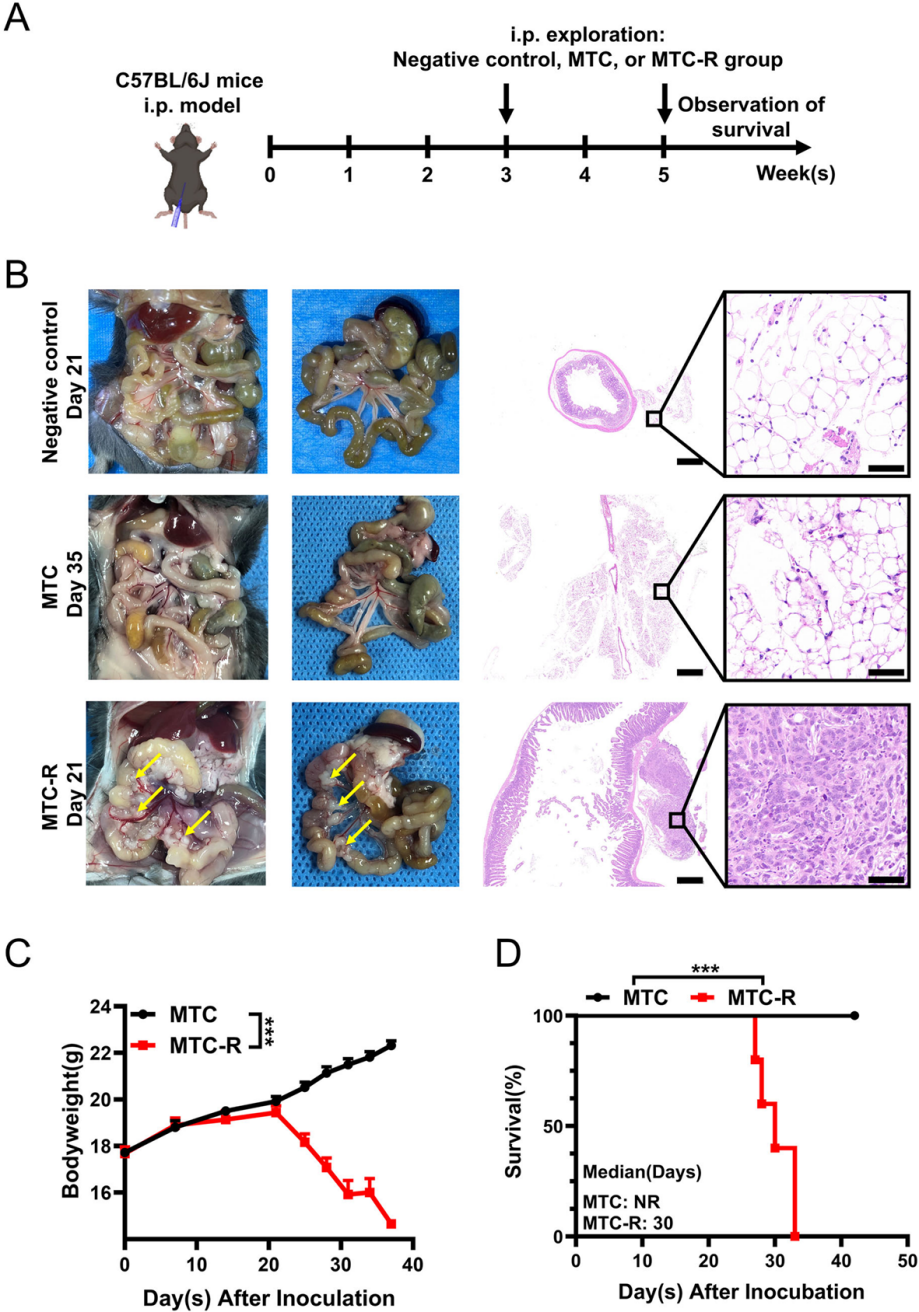
GCPM model of MTC and MTC-R cells in C57BL/6 J mice. **A** Experimental scheme of the MTC and MTC-R i.p. C57BL/6 J mouse models. **B** No visible peritoneal metastatic lesions were observed in the MTC group at the end of day 35. In contrast, diffuse peritoneal metastatic lesions (yellow arrow) were observed within day 21 via H&E staining. The scale bar of H&E staining was 500 μm (left) and 50 μm (right). **C** Comparison of body weight between the MTC and MTC-R groups. **D** Overall survival in the MTC and MTC-R groups. The data are shown as the mean ± SEM. ***: *P* < *0.001*; NR: not reached; more than 50% of the mice survived at the end of the study, and the median survival time was not reached

PM represents the most frequent form of recurrence or metastasis in GC patients and is associated with dismal outcomes (Thomassen et al. 2014). To comprehensively evaluate the PM potential of our cell lines, we established i.p. models and monitored the abdominal manifestations in BALB/c nude mice (Fig. 3A) and C57BL/6 J mice (Fig. 4A). In the i.p. BALB/c nude mouse model, the purtenance and mesentery was clear and no visible tumor was found on day 21 in negative control group, which was confirmed by H&E staining; no visible i.p. metastatic tumours had formed on day 21 in the MTC group, and until 35 days, solid tumours (yellow arrow) in the mesentery and mild localized intestinal dilation were also observed (Fig. 3B). However, in the MTC-R group, diffuse PM lesions (yellow arrow) were clearly observed in the mesentery on day 21, accompanied by localized intestinal obstruction due to the PM (red arrow), which was confirmed by H&E staining (Fig. 3B). Additionally, MTC-R PM model mice presented significantly lower body weight (Fig. 3C) and shorter overall survival (Fig. 3D) than those in MTC PM model mice.

In the i.p. model of C57BL/6 J mice, the purtenance and mesentery was clear and no visible tumor was found on day 21 in negative control group, which was confirmed by H&E staining; no visible PM lesions were observed in the MTC group on day 35, with normal mesenteric morphology, which matched the H&E staining results (Fig. 4B). Conversely, in the MTC-R group, numerous diffuse visible solid tumours in the mesentery were detected on day 21 and confirmed by H&E staining (Fig. 4B). Moreover, a significant sharp decrease in body weight occurred from Day 21 to the experimental endpoint in the MTC-R group; conversely, a steady increase in body weight was observed in the MTC group (Fig. 4C). Compared with the MTC group, the MTC-R group had significantly shorter overall survival (Fig. 4D). The median survival duration was 30 days in the MTC-R group, with no mortality observed in the MTC group during the experimental period. In addition, various intra-abdominal complications, including diffuse mesenteric haemorrhage, gallbladder necrosis, haemorrhagic ascites, intestinal obstruction, necrosis, and gallbladder enlargement caused by biliary obstruction, were also observed in the MTC-R group (Fig. S2C‒E) and confirmed by H&E staining (Fig. S2F, G). Taken together, these results demonstrate that MTC-R cells exhibited greater intra-abdominal tumorigenicity than MTC cells in both immunodeficient and immune-competent mice.

### The ECM-Receptor interaction pathway is aberrantly activated in MTC-R cells

**Fig. 5:**
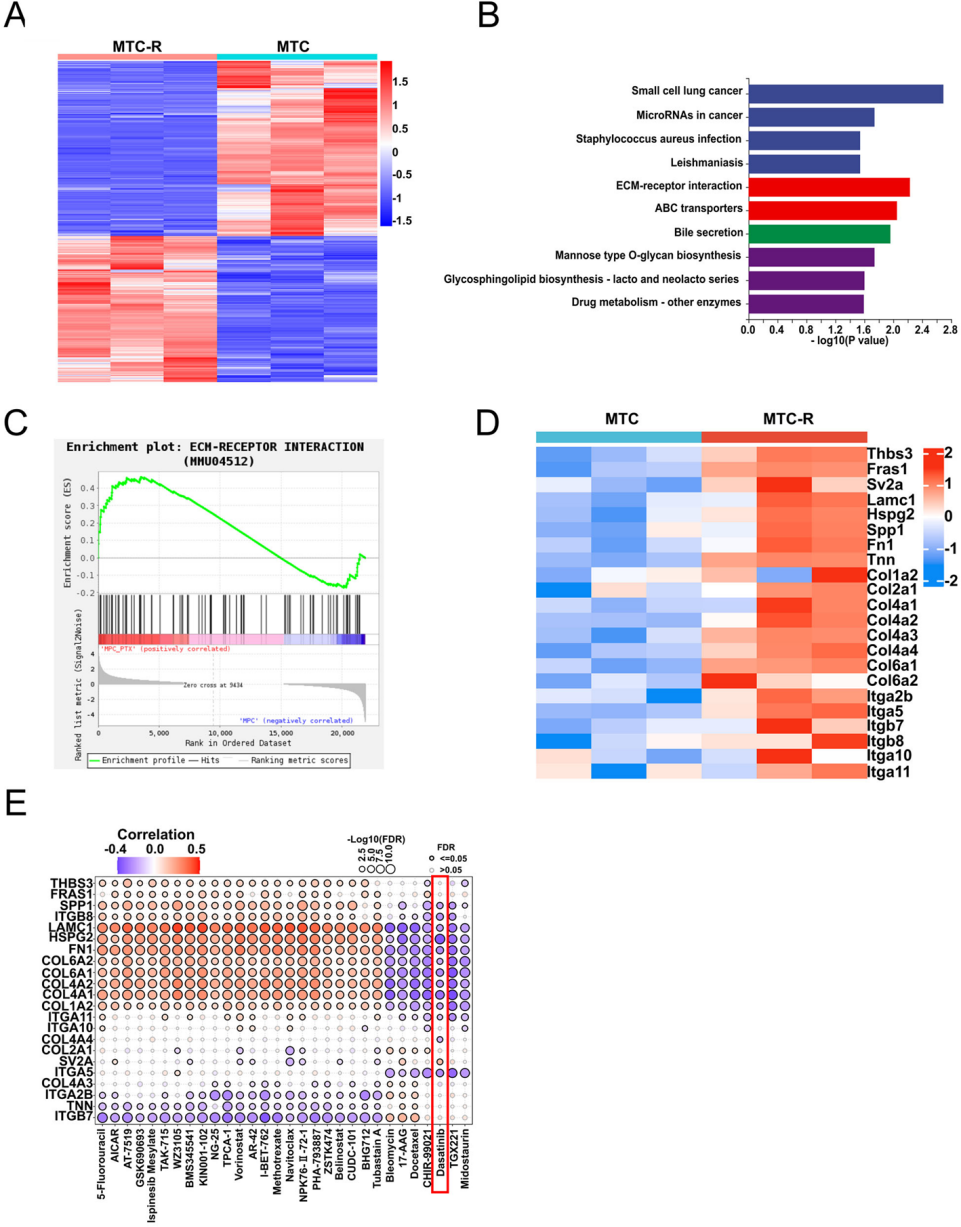
The ECM‒receptor interaction pathway is associated with paclitaxel resistance and high potential for peritoneal metastasis. **A** Heatmap showing genes that are differentially expressed between MTC and MTC-R cells. **B** KEGG enrichment analysis showing the top 10 pathways associated with upregulated genes associated with PTX resistance. **C** GSEA revealed that the ECM‒receptor interaction signalling pathway was upregulated in MTC-R cells. **D** Heatmap outlining the expression of ECM‒receptor interaction pathway-related genes among MTC and MTC-R cells. **E** Prediction of drugs targeting the ECM‒receptor interaction pathway via the GDSC database

To investigate the underlying mechanisms of the high potential for PM and PTX resistance in MTC-R cells, we utilized transcriptome sequencing to detect differential RNA expression between MTC and MTC-R cells. A total of 526 upregulated genes and 629 downregulated genes in MTC-R cells compared with MTC cells according to the heatmap (Fig. 5A) and volcano plot (Fig. S3A). Treatments targeting these upregulated genes may represent new therapeutic options for drug therapy following PTX resistance. Thus, we further assessed the upregulated genes in MTC-R cells to uncover the biological mechanisms underlying the PTX resistance through KEGG enrichment analyses. KEGG analysis revealed that pathways such as extracellular matrix (ECM) interactions, ABC transporters, bile secretion, mannose-type O-glycan biosynthesis, and drug metabolism were significantly enriched in MTC-R cells (Fig. 5B). Previous studies have highlighted the crucial role of ECM‒receptor interactions in cancer dissemination within the tumour microenvironment (Chen et al. 2021; Conklin et al. 2018; Han et al. 2016). Our GSEA results also revealed significant upregulation of the ECM‒receptor interaction pathway (normalized enrichment score (NES) = 1.322, *P* = *0.045*) in MTC-R cells (Fig. 5C). Genes involved in the ECM‒receptor interaction, such as *Lamc1, Spp1, Fn1, Col1a2, Col2a1, Col4a1, Col4a2, Col4a3, Col4a4, Col6a1, Col6a2, Itga2b, Itga5, Itga10, Itga11, Itgb7,* and *Itgb8*, were upregulated significantly in the MTC-R cells (Fig. 5D). Therefore, novel drugs that can target this pathway are urgently needed.

We utilized the online GDSC and CTRP databases to identify potential drugs that may effectively target the ECM-receptor pathway enriched in MTC-R cells. The top 30 predicted drugs from the CTRP database were found to be ineffective against MTC-R cells (Fig. S3B). Analysis of the GDSC database indicated that MTC-R cells may be insensitive to some drugs, such as 5-fluorouracil, vorinostat, and CUDC-101 (Fig. 5E). Similarly, bleomycin, 17-AAG, and docetaxel were positively correlated with *Itga2b*, *Col4A3*, *Tnn,* and *Itgb7* gene expression levels, which indicated a high risk of drug resistance. Dasatinib (DASA), CHIR-99021, TGX221, and Midostaurin exhibited high potential in dealing with PTX resistance. Cell viability assay demonstrated the superb anti-tumor efficacy of DASA in MTC-R, compared with CHIR-99021, TGX221, and Midostaurin (Fig. S4A). Meanwhile, the proliferation and migration of MTC-R was inhibited by DASA in a dose-dependent manner (Fig. S4B, C). DASA, a potent Src inhibitor, may specifically disrupt the ECM-receptor interaction pathway, thereby inhibiting tumour cell proliferation and disease progression (Alhalabi et al. 2022; Mazharian et al. 2023; Wichaiyo et al. 2021; Zhang et al. 2024). Furtherly, we established a MTC-R *Src* knock-down cell line MTC-R-*shSrc* (and its control MTC-R-*shNC*) by *shSrc* (and *shNC*) (Fig. S5A) and evaluated the GCPM tendency between MTC-R-*shNC* and -*shSrc* in PM model mice. The result indicted that the amount of solid tumour metastatic lesions in *shScr* group was quite less than that in *shNC*, especially in mesentery and spleen (Fig. S5B, C). Meanwhile, the MTC-R s.c. tumor mice models were established in C57BL/6 J to evaluate the efficacy of DASA in vivo. The result showed that the growth of MTC-R tumour was inhibited significantly (Fig. S5D-F).

Overall, transcriptome analysis revealed that activation of the ECM‒receptor interaction pathway plays a crucial role in PTX-resistant GCPM. Drug sensitivity prediction on the basis of upregulated genes in the ECM-receptor pathway suggested that DASA, a Src inhibitor, may be a potential drug for the treatment of PTX-resistant GCPM.

### DASA prolonged the survival of MTC-R peritoneal metastasis mice

**Fig. 6:**
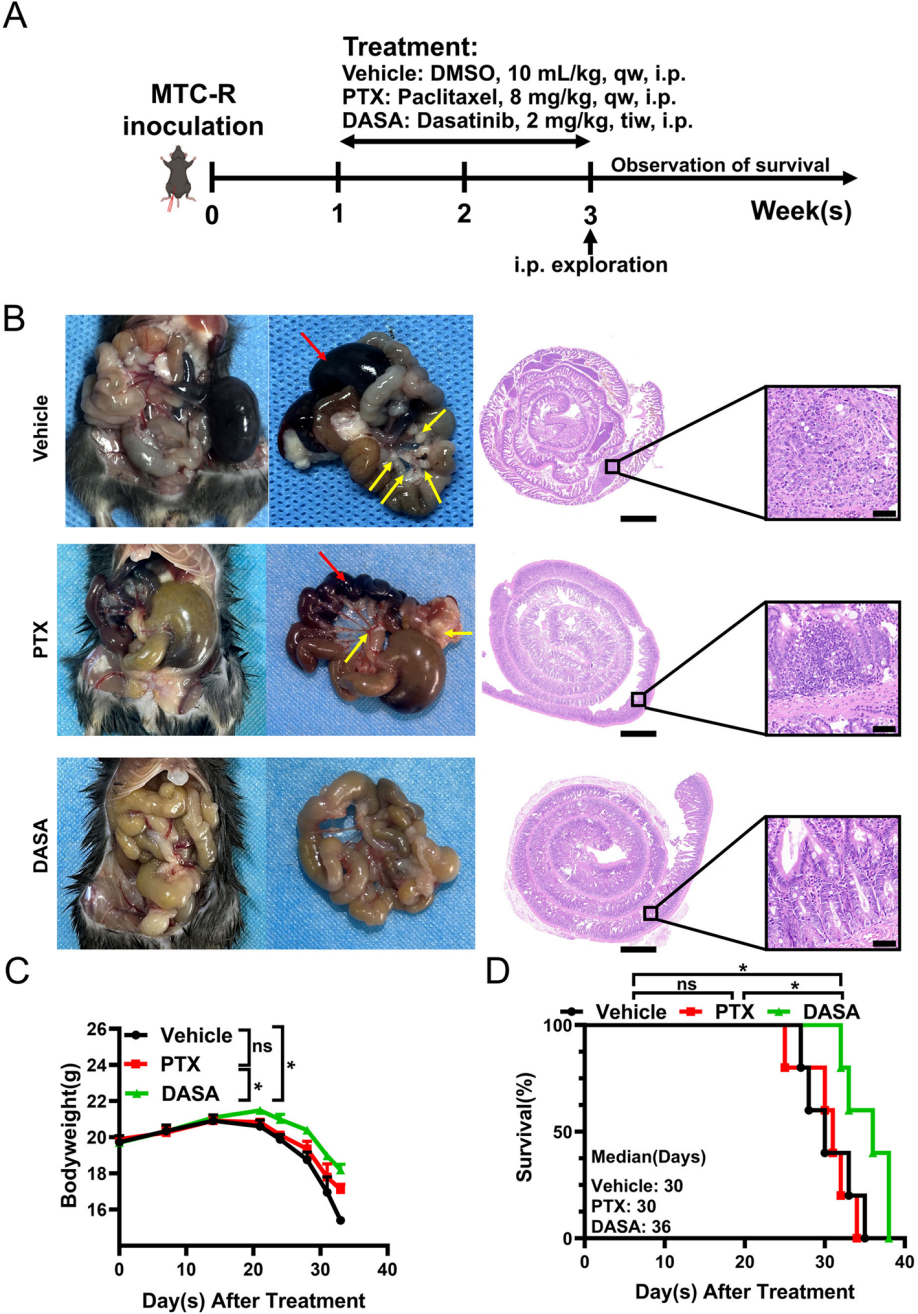
Dasatinib effectively ameliorated disease progression and prolonged overall survival in the MTC-R GCPM C57BL/6 J model. **A** Schematic diagram of in vivo treatment with DASA in the MTC-R peritoneal metastasis model. **B** Obvious mesenteric metastatic lesions (yellow arrow) and intestinal necrosis (red arrow) occurred in the vehicle group and PTX group at 21 days. DASA decreased the incidence of peritoneal metastasis and intestinal obstruction. The scale bar of H&E staining was 1000 μm (left) and 50 μm (right). **C** Changes in body weight among the different groups. **D** Overall survival in different groups. The data are shown as the mean ± SEM. *: *P* < *0.05*; ns: not significant. PTX: paclitaxel; DASA: dasatinib; qw: once weekly; tiw: three times weekly

The above results indicated that DASA has potential antitumour efficacy in MTC-R cells and s.c. tumour mice. Thus, we further evaluated the preclinical efficacy of DASA in the MTC-R PM C57BL/6 J mouse models. The established GCPM mouse models were randomly and equally assigned into three groups (Vehicle, PTX, and DASA) based on the randomized number table. PTX treatment was the reference group for evaluating efficacy (Fig. 6A). Upon abdominal examination and H&E staining, both the vehicle group and the PTX group presented severe PM (yellow arrow) on day 21, with intra-abdominal complications such as intestinal obstruction and necrosis (red arrow) (Fig. 6B). In contrast, the DASA effectively reduced the incidence of abdominal metastasis and associated intra-abdominal complications, preserving the integral structure of mucous glands after 3 cycles of treatment (Fig. 6B). No therapeutic benefit was observed in the PTX group compared with the vehicle group in terms of either body weight (*P* = *0.508*) (Fig. 6C) or overall survival (median survival 31 vs. 30 days, *P* = *0.742*) (Fig. 6D). However, compared with vehicle treatment and PTX treatment, DASA treatment significantly ameliorated body weight loss (Fig. 6C) and prolonged overall survival (Fig. 6D). These results suggest that DASA may serve as a strategy to ameliorate clinical manifestations, delay disease progression, and prolong overall survival in PTX-resistant GCPM treatment.

## Discussion

Recent advances in tumour immunology and immunotherapy-based regimens have revealed promising prospects in the treatment of GC, especially for GCPM (Xu et al. 2023). However, GC patients who are diagnosed with genomically stable or epithelial‒mesenchymal transition molecular subtypes often derive minimal benefit from immunotherapy and chemotherapy (Chao et al. 2021; Yu et al. 2024). The common genetic feature of these two GC molecular subtypes is the loss of *CDH1* expression. GC patients with *CDH1* deficiency typically present with DGC with a high risk of PM, a “cold” tumour microenvironment, and a poor prognosis. Whole-exome sequencing analysis of primary tumours and peritoneal metastatic lesions indicated that these metastatic lesions had a higher frequency of *TP53* and *CDH1* mutations and a lower response rate to chemotherapy (Wang et al. 2020). Therefore, establishing a MGA cell line with *Trp53* and *Cdh1* mutations is crucial for studying this type of GC. Yamamoto previously established mouse GC cell lines that exhibit varying tumorigenic potential in C57BL/6 J mice, providing novel tools for analysing candidate immunotherapies (Yamamoto et al. 2018). However, these mouse GC cell lines only presented a *Trp53* loss-of-heterozygosity background.

Organoids, as tumour mimics, generally inherit the heterogeneity of parental tumours and are widely used in cancer research. In our study, we utilized organoid technology to generate mouse gastric cancer *tc*^*−/−*^ organoids and successfully established an MGA cell line derived from subcutaneously transplanted *tc*^*−/−*^ organoids. We verified the glandular epithelial origin of the MTC cell line via pan-CK IHC staining and confirmed its similarity to organoids by detecting the loss of expression of E-cadherin and p53. Additionally, we verified the loss of function of p53 via a nutlin-3 tumour cell suppression test. Compared with the cell lines established by Yamamoto and other research groups (Nockel et al. 2006; Yamamoto et al. 2018), the MTC was characterized not only by loss of function of p53 but also by a *Cdh1* mutation background. The uniqueness of MTC cells was verified by comparison with other cell banks through STR profiling. We further demonstrated that both MTC and MTC-R cells exhibited different oncogenic potentials in s.c. models of immunodeficient and immune-competent mice. The establishment of MTC cells will facilitate the study of mechanisms and biological processes in *TP53-* and *CDH1*-associated GC. To our knowledge, the MTC is the first cell line to concurrently harbour *Trp53* and *Cdh1* mutations. To date, numerous mouse gastric cancer organoids with specific molecular characteristics have been established via gene editing technology or in genetically engineered mice (Seidlitz et al., 2019). We propose that this study on the establishment of a MGA line derived from mouse gastric cancer organoids subcutaneously transplanted tumours model provides a methodological reference for the generation of additional MGA cell lines with various molecular profiles.

Recent advances in chemoimmunotherapy in patients with GCPM revealed that nonresponder patients presented with increased frequencies of *TP53* and *CDH1* variations (Wang et al. 2020; Yu et al. 2024). These studies highlight that molecular events resulting from the loss of function of *TP53* and *CDH1* are critical factors contributing to aggressive biological behaviour and drug resistance. Since PTX is widely used in second/third-line treatment for GC and is administered intravenously or intraperitoneally (Bonnot et al. 2019; Ishigami et al. 2018; Ishigami et al. 2017; Ishikawa et al. 2020; Yoshikawa et al. 2019), selecting drugs post-PTX resistance in GCPM poses a significant challenge. Thus, we induced the stable PTX-resistant cell line MTC-R through long-term exposure to the pressure of PTX. Compared with MTC cells, MTC-R cells exhibited a more aggressive growth pattern and high potential for PM in mouse models, especially in the i.p. model in C57BL/6 J mice. A major cause of death in patients with GCPM is abdominal complications resulting from metastasis. Our cell lines fairly reproduce the clinical manifestations associated with PM, including intestinal obstruction, ascites, and intestinal necrosis, in the i.p. mouse model. Hence, these cell lines may serve as excellent tools for studying the mechanisms related to GCPM and selecting new drugs, particularly in the context of PTX resistance.

GC cells undergo complex biological processes during PM. Stephen Paget’s “seed and soil” hypothesis suggests that the formation of the PM depends on the interaction between tumour cells and the peritoneal microenvironment (Fidler 2003). Numerous studies have confirmed that the interaction between tumour cells and the ECM plays a crucial role in tumour progression and drug resistance (Huang et al. 2021; Kalli et al. 2023; Mohan et al. 2020). Huang demonstrated via single-cell sequencing technology that chemotherapy-based (containing PTX regent) treatment induced highly plastic clusters of cells to develop into highly proliferative tumour clusters (Huang et al. 2023). These highly proliferative tumour cells are characterized by the activation of pathways, including ECM‒receptor interactions, Hedgehog signalling, and tight junctions. Investigators have revealed that a high level of the ECM signature is correlated with a poor prognosis, drug resistance, and a high risk of GCPM (Chen et al. 2021; Han et al. 2016; Shen et al. 2023). In our study, transcriptome analysis revealed that aberrantly activated ECM‒receptor interaction signalling pathways were intimately associated with PTX resistance and high potential for the PM of MTC-R cells. Upregulated genes enriched in the ECM‒receptor interaction pathway were involved in extracellular collagen production, intercellular adhesion, and cell‒cell communication. These findings suggest that the interaction between tumour cells and the ECM enables tumour cells to acquire metastatic potential and drug resistance. Since the ECM plays crucial roles in tumour progression, drug resistance, and the immunosuppressive microenvironment, therapies targeting the ECM-receptor signalling pathway have been proposed (Pang et al. 2023; Sleeboom et al. 2024). The MTC-R cell line, characterized by *Trp53* and *Cdh1* loss and ECM-Interaction pathway activation, closely recapitulate the molecular and functional features of human diffuse-type gastric cancer, particularly its propensity for peritoneal metastasis (PM). Diffuse-type GC, classified as the genomic stable subtype according to TCGA molecular subtypes, frequently exhibits *TP53* and *CDH1* mutations and is highly associated with PM in human (Cancer Genome Atlas Research 2014). Huang et al. used single-cell analyses further highlight the critical role of ECM-Interaction pathway activation during the transition from human primary gastric lesions to peritoneal dissemination (Huang et al. 2023). In all, similar molecular features were found between *Trp53*^*−/−*^*Cdh1*^*−/−*^ mouse-derived organoids or cell lines and human GC PM according to previous reports and our data. The MTC-R cell line, with its genetic alterations and PM potential, serves as a valuable tool for GC metastasis research, providing deeper insights into the molecular mechanisms driving PM and enhancing the utility of these models for preclinical research and therapeutic development targeting GC metastasis.

To explore drugs that target the ECM‒receptor interaction pathway further, we utilized the online GSCA database to predict drug responses to interfere with this pathway. Most of the drugs predicted in the CTRP database were resistant to PTX, whereas the predictions from the GDSC indicated that CHIR-99021, DASA, TGX221, and midostaurin may be effective against PTX-resistant GCPM. A literature review revealed that DASA is a promising drug for overcoming drug resistance induced by the ECM in glioblastoma and breast cancer (Xiao et al. 2020; Zhang et al. 2024). According to analysis of genomic data, investigators have predicted that DASA can interfere with the ECM-receptor pathway and exert an antitumour effect in chemotherapy-resistant GC (Wu et al. 2024; Zhao et al. 2023). In vitro studies have confirmed that DASA can effectively inhibit the proliferation of GC cell lines (Bertotti et al. 2010; Lee et al. 2022). However, in vivo studies that demonstrate the efficacy of DASA in GC, particularly in the context of GCPM, are lacking. Our study suggested that DASA could delay disease progression and prolong the overall survival of a PTX-resistant GCPM mouse model. These results demonstrate that DASA offers a novel treatment option for overcoming PTX resistance in patients with GCPM characterized by *TP53* and *CDH1* mutation backgrounds. Further studies are needed to investigate the mechanisms underlying the antitumour efficacy of DASA and to determine their clinical applicability in the treatment of GCPM.

In conclusion, we successfully established an MTC cell line originating from *tc*^*−/−*^ organoids with *Trp53* and *Cdh1* mutations and verified its uniqueness via STR profiling. Through long-term exposure to PTX, we generated a corresponding PTX-resistant cell line, MTC-R. Compared with MTC cells, MTC-R cells demonstrated greater potential for tumorigenicity in s.c. and i.p. models in BALB/c nude mice and C57BL/6 J mice. We revealed that the activation of the ECM‒receptor interaction pathway is a key mechanism underlying the high invasive potential associated with PTX resistance. On the basis of drug predictions related to this aberrantly activated pathway, we found that DASA may be an effective treatment for patients with PTX resistance. We further confirmed its biological antitumour efficacy in a PTX-resistant, *Trp53-* and *Cdh1-*deficient GCPM model. These results provide promising options for GCPM treatment following PTX resistance.

## Materials and methods

### Mouse gastric cancer organoid culture

The *Trp53*^*−/−*^*Cdh1*^*−/−*^ (*tc*^*−/−*^) mouse GC organoid was provided by Professor Liyu Huang from Shanghai Jiao Tong University. Previously, normal wild-type gastric organoids (WT) were isolated from *Trp53*^*flox/flox*^*;Cdh1*^*flox/flox*^*;Mist1*-CreER C57BL/6 J mice. The *Trp53*^*flox/flox*^*;Cdh1*^*flox/flox*^ organoids were treated with tamoxifen (TargetMol, T4420) to generate *Trp53*^*−/−*^*Cdh1*^*−/−*^ mouse GC organoids. When the organoids reached 100 μm in diameter, they were passaged. Organoids were washed with PBS and mechanically dissociated by pipetting. Organoids were digested in TrypLE™ Express (Thermo, 12,604,021) at 37 °C for 8~15 min, depending on their size. After digestion, the organoids were centrifuged at 400 × g for 5 min and then washed with organoid culture media (the detailed components of the organoid medium are listed in Table S1). The supernatant was discarded, and the organoids were resuspended in a 1:1 mixture of organoid medium and Matrigel (Corning, 356,231). Next, 50 μL of the suspension was added to a 6-well plate dropwise, with 6 drops/well. All the organoids were cultured at 37 °C in a humidified incubator with 5% CO_2_.

### Animals and ethics

Four- to six-week-old female C57BL/6 J and BALB/c nude mice were provided by Shanghai Chengxi Biotechnology Co., Ltd. The animal studies were approved by the Institutional Animal Care and Use Committee of Shanghai Jiao Tong University (Approval Number: 20221035).

### Isolation of gastric adenocarcinoma cells from >*tc*^*−/−*^ organoid subcutaneously transplanted tumour

We established a MGA cell line derived from *tc*^*−/−*^ organoids transplanted s.c. tumours. 4 × 10^6^ cells (approximately 5 × 10^5^ organoids) with 50% Matrigel were subcutaneously inoculated in the right flank of BALB/c nude mouse. Tumor length and width were measured weekly using calipers. Tumor volume was calculated using the following formula: V = 1/2 × a × b × b, where V = tumor volume, a = maximum tumor diameter, and b = minimum tumor diameter. When the tumour volume reached 200 mm^3^, the BALB/c nude mice were sacrificed, and the s.c. tumours were harvested. The tissue was washed with ice-cold PBS and minced with fine scissors. The tissue fragments were washed with precooled PBS 15 times using a 10 mL pipette in a sterilized clean bench. After washing, the tube was left until the precipitate settled to the bottom, and the supernatant was discarded. Fifty millilitres of EDTA (2 mM) were added to digest the tissue fragments into tumour fragments for 30 ~ 60 min. The tumour units were washed with approximately 20 mL 0.1% BSA in PBS approximately 15 times and then passed through a 70 μm cell strainer (BIOFIL, CSS-013–070). The filtrate was transferred to a 15 mL centrifuge tube, washed with PBS, and centrifuged at 400 × g for 5 min. The cells were resuspended in RPMI 1640 medium (Gibco, 61,870,036) supplemented with 10% FBS (Gibco, A5669701) and 1% antibiotics (Gibco, 15,070,063) and seeded in a dish. If the cell growth conditions were suboptimal, 1:1 organoid medium was added to the medium in a timely manner. After 10 cycles of passage, isolated single cells were cultured in individual wells of a 96-well plate to allow for monoclonal expansion. The monoclonal cells showing the most robust proliferation ability were selected for further studied and the Mouse gastric adenocarcinoma with *Trp53* and *Cdh1* deficiency cell line was established, namely MTC.

### Cell culture

All the cell lines were isolated by our center. Cells were maintained in RPMI 1640 medium (Gibco, 61,870,036) supplemented with 10% FBS (Gibco, A5669701) and 1% antibiotics (Gibco, 15,070,063). The cells were maintained in a humidified incubator with 5% CO_2_ at 37℃.

### Cell growth on glass coverslips

Pre-washed sterilized glass coverslips (CITOTEST 80346–0910) were placed on a dish by predisposing tiny drops of 50 μL medium and the dish was incubated for 30 min. Cells were then seeded at a density of 2 × 10^6^ cells/well in the 6-well plate and allowed to attach for overnight. Aspirating the culture medium, the dish was wash twice with PBS. The cells attached glass coverslips were fixing with 4% paraformaldehyde (Solarbio, P1110) for 30 min. Hematoxylin–eosin staining and immunohistochemistry on cells attached glass coverslips were performed as following procedures.

### Histology and immunohistochemistry

The cell glass coverslips, organoids, and tumours were fixed with 4% paraformaldehyde (Solarbio, P1110), followed by dehydration and then rehydrated and subjected to hematoxylin–eosin staining (H&E) and immunohistochemistry (IHC) using standard procedure. For the H&E staining, the sections were stained with hematoxylin and eosin (Biosharp, BL700A). For IHC, the following primary antibodies were used: p53 (Proteintech, 21,891–1-AP, 1:100), E-cadherin (FineTest, FNab02617, 1:800), pan-CK (Proteintech, 26,411–1-AP, 1:1000).

### Western blotting (WB) analysis

The RIPA lysis buffer was used to extract cells and organoids total proteins. Sodium dodecyl sulfate–polyacrylamide gel electrophoresis (SDS-PAGE) was used to separate extracted proteins. Subsequently, the PDVF membrane (Millipore) was used for protein transfer, following the transferred membrane were incubated with antibodies for overnight at 4℃: GAPDH (Proteintech, 66,002–1-Ig, 1:10,000); p53 (Santa Cruz, sc-126, 1:500); E-cadherin (Proteintech, 20,874–1-AP, 1:1000). The next day, enhanced chemiluminescence reagent (Beyotime, P0018S) was used to detect the antigen–antibody complex on the PDVF membrane.

### Short tandem repeat profiling

Genomic DNA was extracted from MTC cells using the PureLink™ Genomic DNA Extraction Kit (Invitrogen, K182001) following the manufacturer’s instructions. A minimum of 100 ng of genomic DNA was submitted to Shanghai Personal Biotechnology Cp. Ltd. for establishing the DNA profile. Night short tandem repeat (STR) loci (18–3 (FAM), 12–1 (FAM), 4–2 (FAM), 5–5 (FAM), 6–7 (FAM), X-1 (FAM), 9–2 (NED), 15–3 (NED), and 6–4 (NED)), along with the homocontaminated loci D4S2408, were all amplified using Applied Biosystems 3730XL Genetic Analyzer platform (Thermo Fisher Scientific). The genotype results were analysed via GeneMapper™ 6 software (Thermo Fisher Scientific) and compared with STR data from the American Type Culture Collection (ATCC), the German Collection of Microorganisms and Cell Cultures (DSMZ), the Japanese Collection of Research Bioresources (JCRB) and Rikagaku Kenkyūjo (RIKEN).

### Validation of *Cdh1* knockout

*Cdh1* knockout in mouse gastric cancer *tc*^*−/−*^ organoids and MTC cells was confirmed via Sanger sequencing by comparison of the cDNA of these samples with normal WT organoids cDNA. The primers used for *Cdh1* are described in Table S2.

### Cell counting kit-8 (CCK-8) assay

The half-maximal inhibitory concentration determination, cell proliferation, and cell viability assay were involved in CCK-8 assay method. Briefly, cells were allowed to attach overnight at a density of 3,000 cells/well in a 96-well plate, and exposed to the indicated concentration of DMSO, DASA, PTX, CHIR-99021, TGX221, or Midostaurin for the indicated time. The cell medium was replaced with 100 μL fresh medium containing 10 μL CCK-8 solution, and cells were incubated at 5% CO_2_ in a humidified incubatr at 37 ℃ for 1~4 h. The absorbance at a wavelength of 450 nm was dected by a microplate reader (Perkin Elmer). The data was calculated by GraphPad Prism 9.

### Generation of paclitaxel-resistant MTC cells

The increasing drug concentration method was employed in this study to induce drug resistance in the cell line. Initially, the primary IC_50_ (pIC_50_) of the parent cell line was determined. When the cells reached 50% confluence, the MTC cells were exposed to the IC_50_ dose of PTX. After 2 days of treatment, the medium was changed to fresh medium for culture for the next 5 days. Every 4 cycles of treatment, the treated PTX IC_50_ (tIC_50_) level of MTC was reassessed. MTC cells were treated with a new cycle of PTX at the tIC_50_ concentration. After approximately 30 cycles of PTX treatment, the drug resistance index (RI), defined as RI = tIC_50_/pIC_50_, exceeded 5, indicating successful establishment of a PTX-resistant cell line, which was named MTC-R. According to the methods mentioned above, MTC-R cells also underwent monoclonal selection, and the IC_50_ of PTX was remeasured.

### Wound healing assy

The MTC-R cells were inoculated into a 6-well plates until reaching confluence. DASA were added at indicated concentration. Cells were wounded by a 10 μL plastic pipette tip to create a “scratch models”. Photograph the scratches at 0 h and 48 h after DASA treatment. The distance that the cells migrated to the wounded area during this time was measured. The results are expressed as migration index (the migration distance of cells in the experimental group relative to the migration distance of cells in the 0.1% DMSO group). The experiment was repeated three times independently.

### Lentiviral shRNA-Mediated Src Knockdown in Cells

The shRNA sequences targeting *Src* in the pLKO.1 vectors were as follows: *shSrc*: sense, 5’-GACAATGCCAAGGGCCTAAAT-3’. Lentiviruses were produced by co-transfecting HEK293T cells with pLKO.1 or pLVX lentiviral plasmids, along with the packaging plasmids psPAX2 and pMD2.G, using a polyethylenimine -based transfection protocol. The packaged virus was added to the MTC-R cell line and infected for 48 h, followed by selection with puromycin to establish a stable knockdown cell line.

### Subcutaneous tumour model

MTC and MTC-R Cells in the logarithmic growth phase were inoculated subcutaneously into corresponding mice. For s.c. tumor-bearing BALB/c nude mice, 4- to 6-week-old female BALB/c nude mice were randomly and equally stratified into two groups (MTC and MTC-R) based on the randomized number table. Each mouse received 4 × 10^6^ cells in 0.1 mL PBS. For s.c. tumor-bearing C57BL/6 J mice, 4- to 6-week-old female C57BL/6 J mice were randomly and equally stratified into two groups (MTC and MTC-R) based on the randomized number table, each mouse was inoculated with 1 × 10^7^ cells in 0.1 mL PBS containing 50% Matrigel. All mice were examined every 7 days, tumor length and width were measured and tumor volume was calculated using the method mentioned above. Finally, mice were sacrificed and the s.c. tumours were harvested for further analysis. For the in vivo efficacy evaluation of DASA in subcutaneous tumor-bearing C57BL/6 J mice, the experimental protocal was conducted as follows: one week after MTC-R s.c. inoculation, the C57BL/6 J mice were randomly and equally stratified into two groups according to tumour volume and bodyweight using a randomized number table (*n* = 5/group). The treatment groups administered 10 ml/kg of DMSO (vehicle), or dasatinib (5 mg/kg) via i.p. five times per week for 5 weeks. Thoughout the experimental period, body weight and tumor size were measured weekly, and the survival status of the mice was monitored and recorded.

### Intraperitoneal metastasis mice model

4- to 6-week-old female BALB/c nude mice and C57BL/6 J mice were randomly and equally stratified into three groups (Negative control, MTC, and MTC-R) based on the randomized number table. A total of 1 × 10^7^ MTC or MTC-R cells in the logarithmic growth phase were i.p. inoculated into BALB/c nude mice and C57BL/6 J mice respectively. Negative control group mice injected i.p. 0.1 mL PBS each mouse. For the surveillance of tumorigenicity, 3 mice from each group were sacrificed randomly for peritoneal exploration on day 21 or 35 after inoculation. Five mice from each group were recorded continuously for changes in body weight and overall survival. For the in vivo efficacy evaluation of DASA, one week after MTC-R i.p. inoculation, the C57BL/6 J mice were randomly and equally stratified into three groups based on the randomized number table (*n* = 8/group) and administered 100 μL of DMSO (vehicle), PTX (8 mg/kg) once a week, or dasatinib (2 mg/kg) via i.p. injection three times per week. The body weight of the mice were recorded weekly, and the survival status of the mice was documented throughout the experiment. On day 21, 3 mice from each group were sacrificed randomly for i.p. exploration. Five mice in each group were monitored continually for changes in body weight and to assess overall survival.

### RNA-seq processing and data analysis

The cells were cultured and harvested via the TRIzol reagent (Invitrogen, 15596018CN) for total RNA extraction following the manufacturer’s protocol. All RNA-seq samples underwent quality control and were customized for analysis by Shanghai Personal Biotechnology Co., Ltd. Detailed procedures for establishing the sequencing libraries are provided in Supplementary materials. Differential gene expression analysis was conducted via DESeq (v1.38.3) with the following filtering conditions: |log2FoldChange|> 1 and P value < 0.05. The R language Pheatmap package (v1.0.12) was used to perform bidirectional clustering analysis of all the DEGs. A heatmap was generated using the Euclidean and Complete Linkage method based on expression levels across different samples. ClusterProfiler (v4.6.0) software was used to analyse the enrichment of enriched KEGG pathways among the upregulated genes, with a focus on pathways with significant enrichment (*P* value < 0.05). The gene set enrichment analysis (GSEA) (v4.1.0) tool was used to conduct enrichment analysis across all genes and generate pathway enrichment maps.

### Prediction of drugs for paclitaxel-resistant cells

The Gene Set Cancer Analysis (GSCA) website (http://bioinfo.life.hust.edu.cn/GSCA) was used to explore the relationships between signature gene sets and drug sensitivity (Liu et al., 2023). The online platform integrates data from the Genomics of Drug Sensitivity in Cancer (GDSC) and Clinical Trails Reporting Program (CTRP) databases. The correlation of PTX resistance-related gene expression with drug sensitivity was next assessed via Spearman correlation analysis. A negative correlation indicates that increased gene expression may increase drug sensitivity. A lower false discovery rate (FDR) indicates greater predictive significance. Ultimately, FDA-approved drugs with both a low FDR and a negative correlation were prioritized for selection.

### Statistical analysis

The data are presented as the means ± standard errors of the means. Continuous variable data were analysed via two-way ANOVA in GraphPad Prism 9. Two-tailed Student’s *t* tests were performed to compare tumour weights via GraphPad Prism 9. Drug concentrations were log-transformed for normalization, and IC_50_ values were calculated via nonlinear regression in GraphPad Prism 9. Overall survival analysis was conducted with GraphPad Prism 9, and survival curves were compared via the log-rank test. A *P* value less than 0.05 was considered to indicate statistical significance.

## Supplementary Information

Supplementary Material 1. Fig. S1: The characteristics of MTC cells and WT/*tc*^*-/-*^ organoids; Fig. S2: The bodyweight of s.c. tumour mice and intra-abdominal complications associated with intraperitoneal metastasis of MTC-R cells in PM mice; Fig. S3: Different gene analysis and drugs prediction; Fig. S4: The prolification and migration of MTC-R was inhibited by Dasatinib; Fig. S5: The effect of *Src* and its inhibitor Dasatinib on MTC-R in vivo; Table S1: The component of mouse gastric cancer organoids medium; Table S2 The specific primers for *Cdh1*; Table S3: Genotyping results of STR and Amelogenin loci in MTC cells; RNA-seq data processing.

## Data Availability

The RNA-seq data have been deposited in the GEO Database: GSE293310. The datasets used and/or analyzed during the current study are available from the corresponding author on reasonable request.

## Abbreviations

GC: Gastric Cancer
PM: Peritoneal Metastasis
GCPM: Gastric Cancer Peritoneal Metastasis
PTX: Paclitaxel
MTC: Mouse *Trp53*^*−*^^*/*^^*−*^* Cdh1*^*−*^^*/*^^*−*^ GC cell line
MTC-R: Paclitaxel-resistant MTC cell line
MGA: Mouse gastric adenocarcinoma
IC_50_: Half Maximal Inhibitory Concentration
RI: Resistance Index
GDSC: Genomics of Drug Sensitivity in Cancer
CTRP: Clinical Trails Reporting Program
H&E: Hematoxylin-Eosin
IHC: Immunohistochemistry
WT: Wild Type
