# Supplementary material for: Dasatinib demonstrates efficacy in organoid derived paclitaxel-resistant Trp53/Cdh1-deficient mouse gastric adenocarcinoma with peritoneal metastasis
Source: Cell Regen. 2025 Apr 29;14:16. doi: 10.1186/s13619-025-00232-2 (PMC12040775; doi:10.1186/s13619-025-00232-2)
Supplement: Supplementary file 1 — Supplementary Material 1. Fig. S1: The characteristics of MTC cells and WT/tc-/- organoids; Fig. S2: The bodyweight of s.c. tumour mice and intra-abdominal complications associated with intraperitoneal metastasis of MTC-R cells in PM mice; Fig. S3: Different gene analysis and drugs prediction; Fig. S4: The prolification and migration of MTC-R was inhibited by Dasatinib; Fig. S5: The effect of Src and its inhibitor Dasatinib on MTC-R in vivo; Table S1: The component of mouse gastric cancer organoids medium; Table S2 The specific primers for Cdh1; Table S3: Genotyping results of STR and Amelogenin loci in MTC cells; RNA-seq data processing. [file 13619_2025_232_MOESM1_ESM.docx]

**Dasatinib demonstrates efficacy in Organoid Derived** **Paclitaxel-resistant *Trp53/Cdh1-*deficient Mouse Gastric Adenocarcinoma with Peritoneal Metastasis**

**Supplementary Figures**

**Fig. S1 The characteristics of MTC cells and WT/*tc^-/-^* organoids. (A**) Representative IHC staining for E-cadherin and p53 in the WT organoid (cale bar, 50 μm). (B) Western blotting revealed the expression of p53 and E-cadherin in organoids and cell line. (C) Sanger sequencing indicated the deletion of *Cdh1*-Exon3 in MTC and *tc^-/-^* organoid. (D) The inhibitory effects of nutlin-3 in MTC cells. (E) The MTC-R cells displayed with spindle shape morphology (scale bar, 20 μm). (F) MTC and MTC-R showed different proliferation potential. Data were shown as mean ± SEM. ***: *P<0.001*; WT: wild type mouse gastric epithelial organoid; *tc^-/-^*: *Trp53-* and *Cdh1-*deficent mouse gastric cancer organoid.


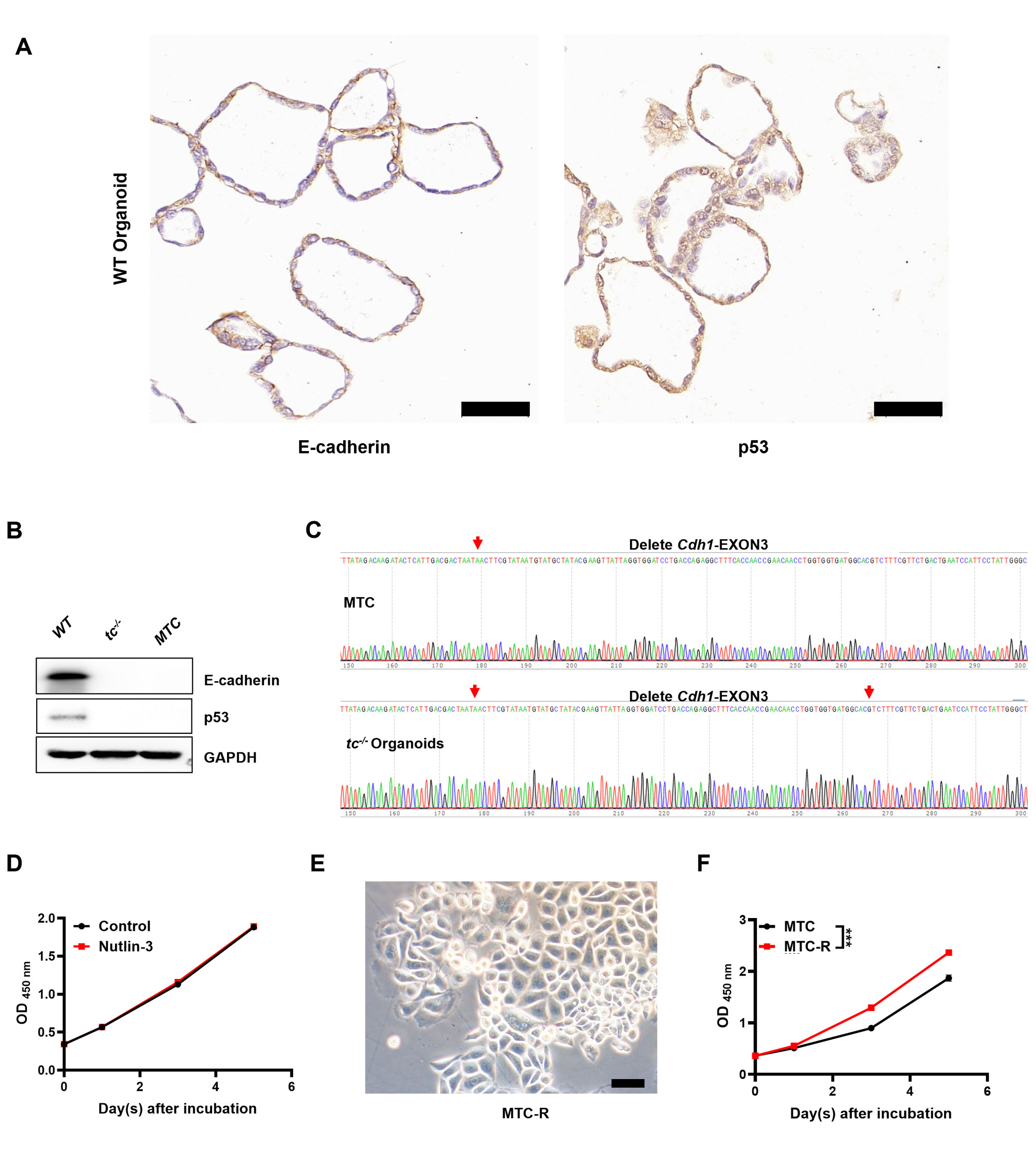


**Fig. S2 The bodyweight of s.c. tumour mice and intra-abdominal complications associated with intraperitoneal metastasis of MTC-R cells in PM mice.** (A-B) The bodyweight of s.c. tumour mice in Balb/c nude and C57BL/6J mice. (C) Diffuse bleeding within the mesentery (blue arrow) and gallbladder enlargement (red arrow) due to MTC-R cells intraperitoneal metastasis. (D) Hemorrhagic ascites in MTC-R peritoneal metastasis mice model. (E) Gallbladder enlargement (red arrow) due to biliary obstruction caused by omental bursa metastasis and intestinal necrosis (blue arrow) caused by mesenteric metastasis. (F) Peritoneal metastasis caused intestinal necrosis. Left: scale bar, 500 μm; Right: scale bar, 50 μm. (G) The occurrence of metastasis in greater and lesser omentum. Left: scale bar, 500 μm; Right: scale bar, 50 μm. s.c.: subcutaneous(ly). ns: no significance


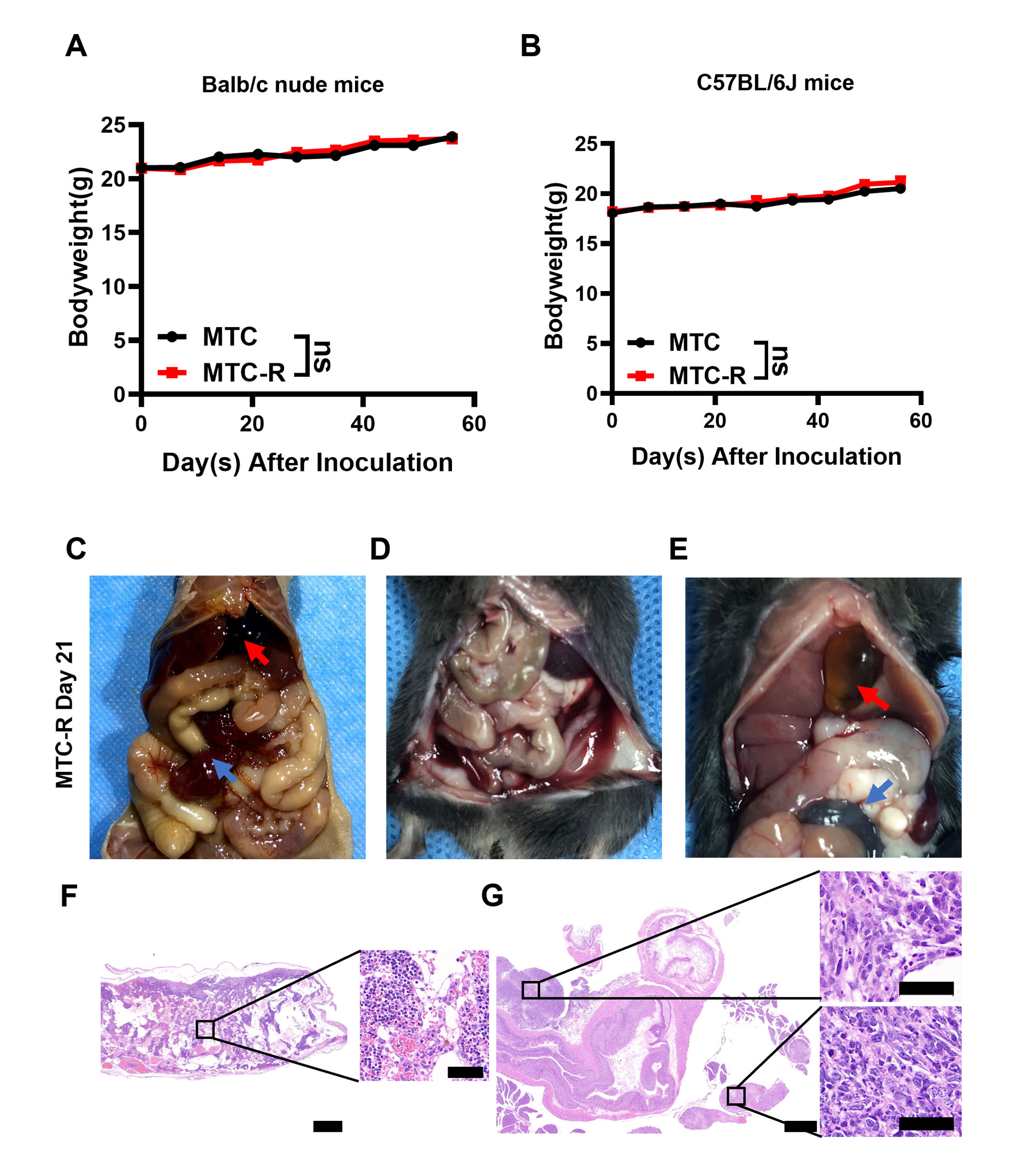


**Fig. S3 Different gene analysis and drugs prediction.** (A) Volcano plot showing genes that are differentially expressed in MTC and MTC-R. (B) Drugs prediction in CTRP database based on different genes correlated with ECM signal pathway.


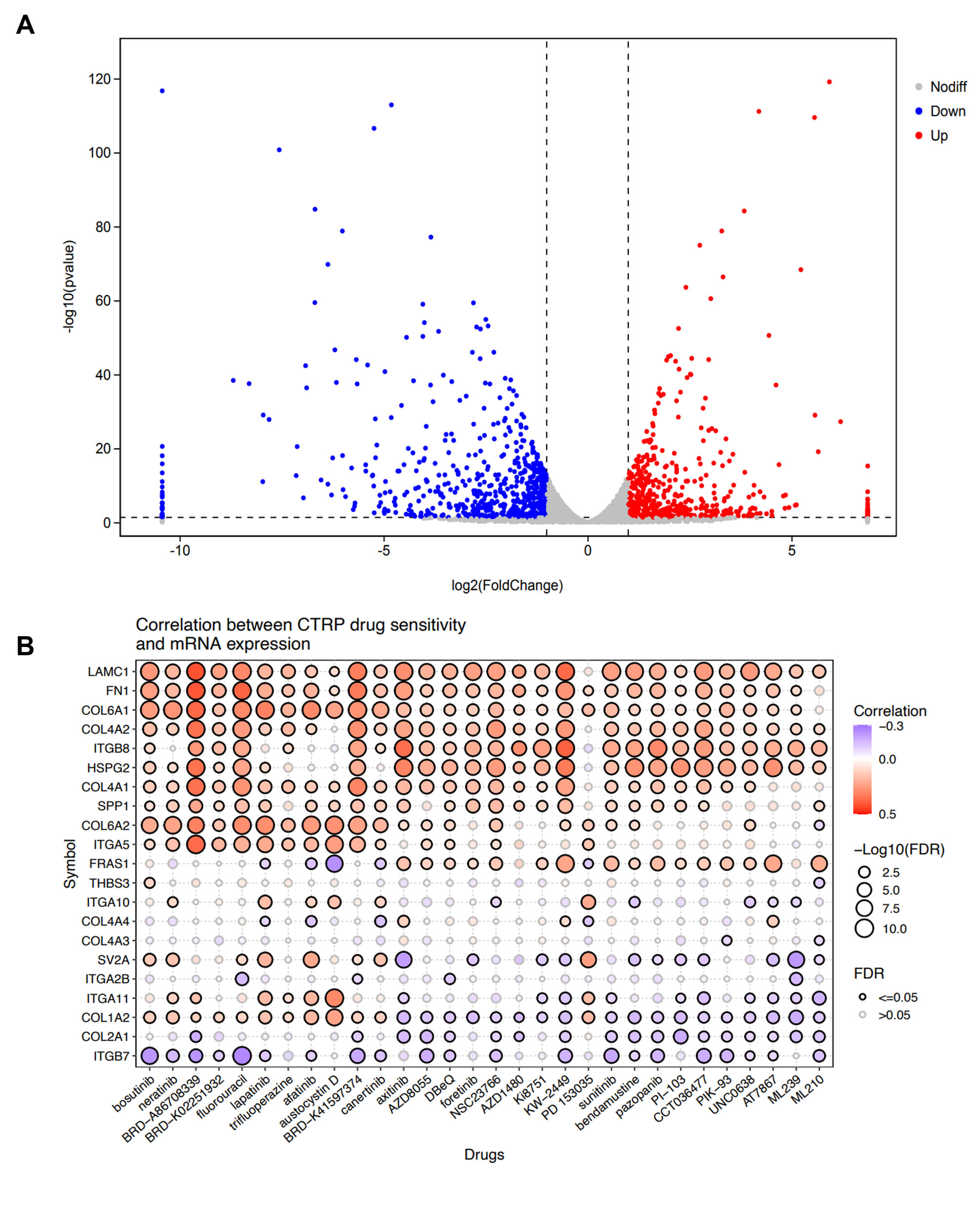


**Fig. S4 The prolification and migration of MTC-R was inhibited by Dasatinib.** (A) The cell viability of MTC-R incubated with 1μM DASA, CHIR-99021, TGX221, or Midostaurin for 48 h. (B) The prolification of MTC-R incubated with indicated DASA (or DMSO). (C) The migration of MTC-R of MTC-R incubated with indicated DASA (or DMSO). Scratch edge is demarcated by the red line. DASA: Dasatinib**.** Scale bar, 200 μm. The data are shown as the mean ± SEM. **: *P<0.01*, ***: *P<0.001*, ****: *P<0.0001*.


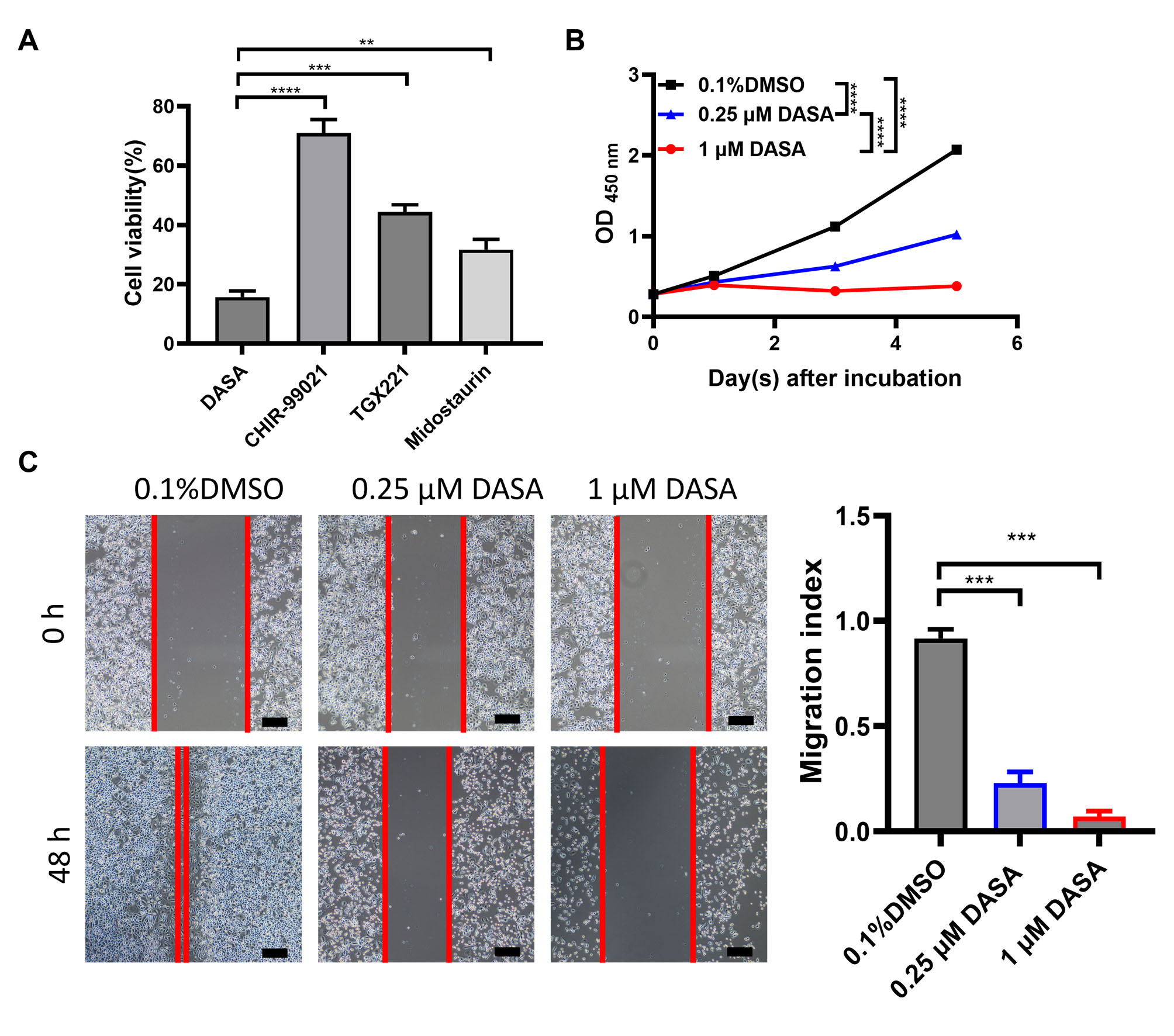


**Fig. S5 The effect of *Src* and its inhibitor Dasatinib on MTC-R in vivo.** (A) Western blotting revealed the expression of Src in MTC-R- *shNC* and -*shSrc* cell line. (B) The diffuse peritoneal metastatic lesions (red arrow) in MTC-R- *shNC* and -*shSrc* group. (C) The amount of metastatic foci in MTC-R- *shNC* and -*shSrc* group(n=5). (D) Schematic diagram of in vivo treatment with DASA in the MTC-R s.c. C57BL/6J mouse models. (E) Curves of tumour volume in the MTC-R s.c. C57BL/6J mouse models(n=5). (F) Gross images of tumours after tumour resection at the endpoint of the experiment in the MTC-R s.c. C57BL/6J mouse models. s.c., subcutaneous(ly). DASA: Dasatinib**.** The data are shown as the mean ± SEM. *: *P<0.05*, ***: *P<0.001.*


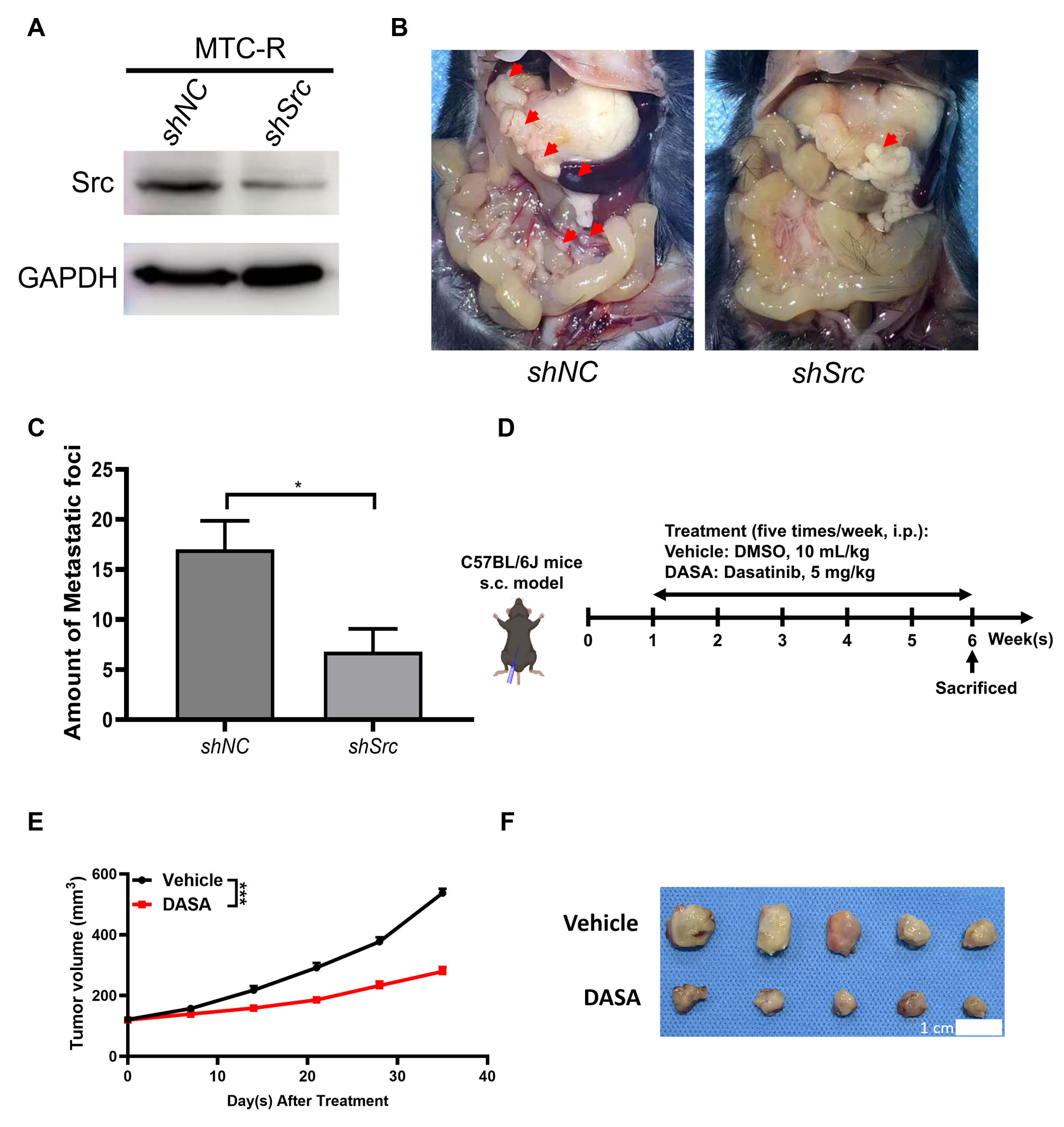


**Supplementary Tables**

**Table S1 The component of mouse gastric cancer organoids medium**

| Regents | Concentration | Manufacturer | Cat# |
| --- | --- | --- | --- |
| Advanced DMEM/F12 | 1 | Thermo | 12634010 |
| Penicillin/Streptomycin | 1× | NCM Biotech | C100C5 |
| GlutaMAX^TM^ Supplement | 1× | Thermo | 35050061 |
| HEPES | 1mM | Thermo | 15630080 |
| B-27 Supplement | 1× | Thermo | 12587001 |
| N-2 Supplement | 1× | Thermo | 17502048 |
| N-Acetyl-L-cysteine | 100nM | Sigma | A9165 |
| Nicotinamide | 400mM | Biogems | 9899208 |
| Wnt-3a | 100µg/ml | Peprotech | 315-20 |
| R-Spondin-1 | 1000µg/ml | Peprotech | 315-32 |
| Noggin | 100µg/ml | Peprotech | 250-38 |
| EGF | 50µg/ml | Peprotech | 315-09 |
| FGF-10 | 100µg/ml | Peprotech | 450-61 |
| Gastrin Ⅰ | 100µM | Peprotech | 1003377 |
| Thiazovivin | 5mM | Peprotech | 122718 |

**Table S2 The specific primers for *Cdh1***

| Primers | Sequence (5’-3’) |
| --- | --- |
| *Cdh1*-EXON3-F | AGGAGCGCCAGGCTTATAGA |
| *Cdh1*-EXON3-R | CTGAGAACCACTGTCGGAGG |

**Table S3 Genotyping results of STR and Amelogenin loci in MTC cells**

| Loci | Detected cell line STR information | | | | Cell bank STR information | | | |  |
| --- | --- | --- | --- | --- | --- | --- | --- | --- | --- |
|  | MTC cell line | | | |  | | | |  |
|  | Allele1 | Allele2 | Allele3 | Allele4 | Allele1 | Allele2 | Allele3 | Allele4 |  |
| 4-2 | 238.55 | ND | ND | ND | ND | ND | ND | ND |  |
|  | [20.3] |  |  |  |  |  |  |  |  |
| 5-5 | 344.65 | 348.66 | ND | ND | ND | ND | ND | ND |  |
|  | [16] | [17] |  |  |  |  |  |  |  |
| 6-4 | 296.79 | ND | ND | ND | ND | ND | ND | ND |  |
|  | [17] |  |  |  |  |  |  |  |  |
| 6-7 | 347.29 | 355.4 | ND | ND | ND | ND | ND | ND |  |
|  | [15] | [17] |  |  |  |  |  |  |  |
| 9-2 | 234.15 | 238.23 | ND | ND | ND | ND | ND | ND |  |
|  | [18] | [19] |  |  |  |  |  |  |  |
| 12-1 | 230.55 | ND | ND | ND | ND | ND | ND | ND |  |
|  | [17] |  |  |  |  |  |  |  |  |
| 15-3 | 201.16 | ND | ND | ND | ND | ND | ND | ND |  |
|  | [22.3] |  |  |  |  |  |  |  |  |
| 18-3 | 152.81 | ND | ND | ND | ND | ND | ND | ND |  |
|  | [16] |  |  |  |  |  |  |  |  |
| X-1 | 409.19 | ND | ND | ND | ND | ND | ND | ND |  |
|  | [27] |  |  |  |  |  |  |  |  |
| D4S2408 | ND | ND | ND | ND | ND | ND | ND | ND |  |
|  |  |  |  |  |  |  |  |  |  |

**ND: not detected.**

**Supplementary materials**

**RNA-seq data processing**

The concentration, quality and integrity of total RNA were determined using a NanoDrop spectrophotometer (Thermo Scientific). 3 μg of RNA were used as input material for the RNA sample preparations. Sequencing libraries were generated according to the following steps. Firstly, mRNA was purified by using poly-T oligo-attached magnetic beads. Fragmentation was carried out using divalent cations under elevated temperature in an Illumina proprietary fragmentation buffer. First and second strand cDNA was synthesized using random oligonucleotides with Super Script II and DNA Polymerase I with RNase H, respectively. Remaining overhangs were converted into blunt ends via exonuclease/polymerase activities and the enzymes were removed. After adenylation of the 3′ ends of the DNA fragments, Illumina PE adapter oligonucleotides were ligated to prepare for hybridization. To select cDNA fragments of the preferred 400-500 bp in length, the library fragments were purified using the AMPure XP system (Beckman Coulter,Beverly, CA, USA). DNA fragments with ligated adaptor molecules on both ends were selectively enriched using Illumina PCR Primer Cocktail in a 15 cycle PCR reaction. Products were purified (AMPure XP system) and quantified using the Agilent high sensitivity DNA assay on a Bioanalyzer 2100 system (Agilent). The original data in FASTQ format (raw data) was generated from the sequencing platform. The fastp (0.22.0) was used to filter the raw date to get high quality sequence for further study. The reference genome and gene annotation files were downloaded from genome website. The filtered reads were mapping to the reference genome using HISAT2 (v2.1.0).
